# Drug-induced QT interval prolongation in patients with heart failure with preserved ejection fraction

**DOI:** 10.1371/journal.pone.0308999

**Published:** 2024-08-19

**Authors:** Chien-Yu Huang, Brian R. Overholser, Kevin M. Sowinski, Heather A. Jaynes, Richard J. Kovacs, James E. Tisdale

**Affiliations:** 1 Department of Pharmacy Practice, College of Pharmacy, Purdue University, West Lafayette and Indianapolis, Indiana, United States of America; 2 Division of Clinical Pharmacology, Department of Medicine, School of Medicine, Indiana University, Indianapolis, Indiana, United States of America; 3 Department of Medicine, Krannert Cardiovascular Research Center, School of Medicine, Indiana University, Indianapolis, Indiana, United States of America; University of Minnesota, UNITED STATES OF AMERICA

## Abstract

Heart failure (HF) with reduced ejection fraction (HFrEF) is a risk factor for drug-induced QT interval prolongation. It is unknown if HF with preserved ejection fraction (HFpEF) is also associated with an increased risk. Dofetilide and sotalol are potent QT interval-prolonging agents that are frequently used in patients with HFpEF, in whom atrial fibrillation is a common comorbidity. We tested the hypothesis that the risk of QT interval prolongation associated with dofetilide and sotalol is increased in patients with HFpEF. We conducted a retrospective cohort study conducted using electronic health records from the Indiana Network for Patient Care (January 31, 2010 –May 3, 2021). After removing patients with overlapping diagnoses of HFpEF and HFrEF, no diagnosis code, and absence of QT interval records, we identified patients taking dofetilide or sotalol among three groups: HFrEF (n = 138), HFpEF (n = 109), and no HF (n = 729). QT prolongation was defined as heart rate-corrected QT (QTc) > 500 ms during dofetilide/sotalol therapy. Unadjusted odds ratios (OR) for QT prolongation were determined by univariate analysis. Adjusted ORs were determined by generalized estimating equations (GEE) with logit link to account for an individual cluster with different times of hospitalization and covariates. QTc prolongation associated with dofetilide or sotalol occurred in 53.2%, 71.7% and 30.0% of patients with HFpEF, HFrEF, and patients with no HF, respectively. After adjusting for age, sex, race, serum potassium and magnesium concentrations, kidney function, concomitant drug therapy, and comorbid conditions, the adjusted odds of QTc prolongation were significantly higher in patients with HFpEF [OR = 1.98 (95% CI 1.17–3.33)], and in those with HFrEF [OR = 5.23, (3.15–8.67)], compared to those with no evidence of HF. The odds of QT prolongation among inpatients receiving dofetilide or sotalol were increased in patients with HFpEF and HFrEF compared to those who did not have HF.

## Introduction

Torsades de pointes (TdP) is a life-threatening ventricular arrhythmia [1, 2] that may be caused by more than 200 drugs on global markets, including commonly used antiarrhythmic drugs, antibiotics, antidepressants, antipsychotic agents, and others [2, 3]. QT interval prolongation on the electrocardiogram (ECG) portends an increased risk of TdP [1, 2]. Heart failure with reduced ejection fraction (HFrEF) is a risk factor for drug-induced TdP [4]. In patients with HFrEF, the incidence of dofetilide-induced TdP is 3.3% [5], compared to < 1% in those without heart failure [6]. The incidence of TdP associated with ibutilide is 1.7–4.1% in patients without heart failure [7–9], but is higher in those with HFrEF [10, 11]. HFrEF is a risk factor for sotalol-induced TdP [12]. We have shown previously that patients with HFrEF demonstrate increased sensitivity to drug-induced QT interval lengthening [13]. Consequently, guidelines recommend avoidance of QT interval-prolonging drugs such as ibutilide in some populations with HFrEF, due to the enhanced risk of TdP [14–16].

Heart failure with preserved ejection fraction (HFpEF) comprises at least 50% of patients with heart failure, and its prevalence continues to increase [17]. Whether patients with HFpEF are at higher risk of drug-induced QT prolongation or TdP is unknown. QT interval prolongation is predictive of diastolic dysfunction [18] which is associated with an increased risk of arrhythmic death or resuscitated cardiac arrest [19]. In an animal model in which HFpEF was induced in rodents by salt loading, QT intervals were prolonged [20] and ventricular arrhythmias occurred more frequently compared to control animals; sudden death occurred in 31% of the rats with HFpEF [20]. Animals with HFpEF exhibited prolonged ventricular action potential duration with multiple reentrant circuits and downregulated I_to_, I_Kr_, and I_K1_ currents [21]. We previously conducted a small pilot study that suggested that patients with HFpEF display enhanced response to drug-induced QT interval lengthening compared to a control group of patients who did not have heart failure [22]. Collectively, these data suggest that HFpEF could be associated with an increased risk of drug-induced QT prolongation and TdP.

Atrial fibrillation (AF) occurs commonly in patients with HFpEF and is one of the primary antecedents and predictors of development of HFpEF [23]. The antiarrhythmic drugs dofetilide and sotalol are used commonly in patients with AF for maintenance of sinus rhythm, and therefore are used commonly in patients with HFpEF [14]. Due to the risk of TdP associated with these drugs, initiation of therapy with dofetilide or sotalol must occur during a Food and Drug Administration-mandated 3-day hospitalization period [24, 25], during which QT intervals are measured and drugs doses are adjusted where necessary. If the QT interval becomes prolonged, the drug may be discontinued. As dofetilide and sotalol are potent QT interval-prolonging drugs that are associated with TdP and are used commonly in patients with HFpEF, assessment of the potential for enhanced risk of QT interval prolongation in this population is important.

In this study, we tested the hypothesis that patients with HFpEF are at increased risk of drug-induced QT interval lengthening compared to patients with no HF.

## Materials and methods

### Data source

Data were obtained using electronic health records (EHR) from the Indiana Network for Patient Care Research database (INPCR), which is managed by the Regenstrief Institute’s Indiana Health Information Exchange (https://www.ihie.org) [26]. The INPCR receives data from over 100 distinct healthcare entities, including hospitals, health networks, and insurance providers, representing over 18 million patients, 10 billion clinical observations, 951 million encounter records, and over 147 million mineable text reports. The INPCR also receives data on drugs that have been prescribed to patients within its institutions. This study followed the Strengthening of Reporting of Observational Studies in Epidemiology reporting guideline for cohort studies [27]. This investigation was granted exempt status by the Indiana University Institutional Review Board.

### Study design and population

We conducted a retrospective cohort study that included patients taking dofetilide or sotalol among three groups: 1) Those with HFpEF, 2) Those with HFrEF (positive control) and 3) Those with no evidence nor history of heart failure (negative control). We identified patients 18 years of age or older taking dofetilide or sotalol during their hospital stay during the period January 31, 2010 through May 3, 2021. To identify patients with HFpEF, HFrEF and patients without a history of heart failure, we used the International Classification of Diseases (ICD)-9 and ICD-10 codes from patients’ diagnosis codes during the hospital stay, as well as comorbidities (S1 Table). Patients having overlapping diagnoses with HFrEF and HFpEF were excluded. In patients not having diagnosis codes of HFrEF and HFpEF, we removed patients with ICD-9 or ICD-10 codes for congestive heart failure (S1 Table) and defined this group as the no heart failure group. Further, we removed patients who had hospitalization records but for whom prescriptions for dofetilide or sotalol were canceled, suspended, or voided. QT intervals in the EHR database were documented from 12-lead ECGs. If a patient had multiple ECGs, we selected that with the longest QTc interval. Patients without any diagnosis records or documented heart rate-corrected QT (QTc) intervals were excluded. Patients hospitalized for more than 31 days or for whom there were no recorded laboratory values during their hospital stay were also excluded.

### Exposure, outcomes and covariates

We identified patient demographics (age, sex, race), comorbidities (hypertension, diabetes, chronic kidney disease, coronary artery disease, myocardial infarction, hyperlipidemia, stroke, chronic obstructive pulmonary disease), laboratory values during hospital stay (serum calcium, potassium, and creatinine concentrations), concomitant drug use during hospital stay [using other drugs known to cause TdP, drugs with a possible risk of TdP, and/or drugs with a conditional risk of TdP (from the QT drugs list at www.crediblemeds.org) [3], calcium channel blockers, loop diuretics, thiazide diuretics, beta-blockers, ACE inhibitors, angiotensin-receptor blockers, digoxin, and aldosterone receptor antagonists]. Age older than 60 and younger than 80 years were categorized into 10-year increments (< = 60, 61 to 70, 71 to 80, and >80 years of age). Sex was grouped into female and male. Race was grouped into three categories: White, Black, and other. Serum potassium <3.5 mmol/L was defined as hypokalemia, and serum creatinine concentration >1.5 mg/dL was defined as elevated serum creatinine. For drug use, we identified whether or not patients used the particular drug during the hospital stay. We excluded prescription ophthalmic ointment and epidermal medications and only included drugs taken orally.

Our primary outcome measure was heart rate-corrected QT (QTc) interval prolongation, defined as QTc > 500 ms during hospitalization while taking dofetilide or sotalol. QT intervals in the datasets were corrected for heart rate using the Bazett’s (96.3%) or Fridericia formulae (3.7%) from the INPCR data source.

### Statistical analysis

Normality of data distribution was assessed using the Kolmogorov-Smirnov and Shapiro-Wilk tests. Baseline characteristics summarized with percentages were compared using the Chi-square test, and those with numerical values were compared using the Kruskal–Wallis test to account for the non-normal distribution of data among HFpEF, HFrEF and the no-HF group. Significance of differences in the proportion of subjects with QTc prolongation was assessed using the Chi-square test. To assess the association of QT interval lengthening and HFpEF, we used generalized estimating equations (GEE) with logit link to account for an individual cluster with different times of hospitalization relative risk in those without HF and calculated unadjusted and adjusted odds ratios for QTc > 500ms. Concomitant drugs were also identified as covariates. GEE with 10,000 replicates were generated to test the validation of the results. In a sensitivity analysis, we used backward selection in our GEE model to select characteristics with a p value <0.25 and bootstrapping with 10,000 replicates to examine the model stability. p values less than 0.05 were considered significant. SAS version 9.4 was used to perform all the analyses.

## Results

### Patient cohort selection

**Fig 1:**
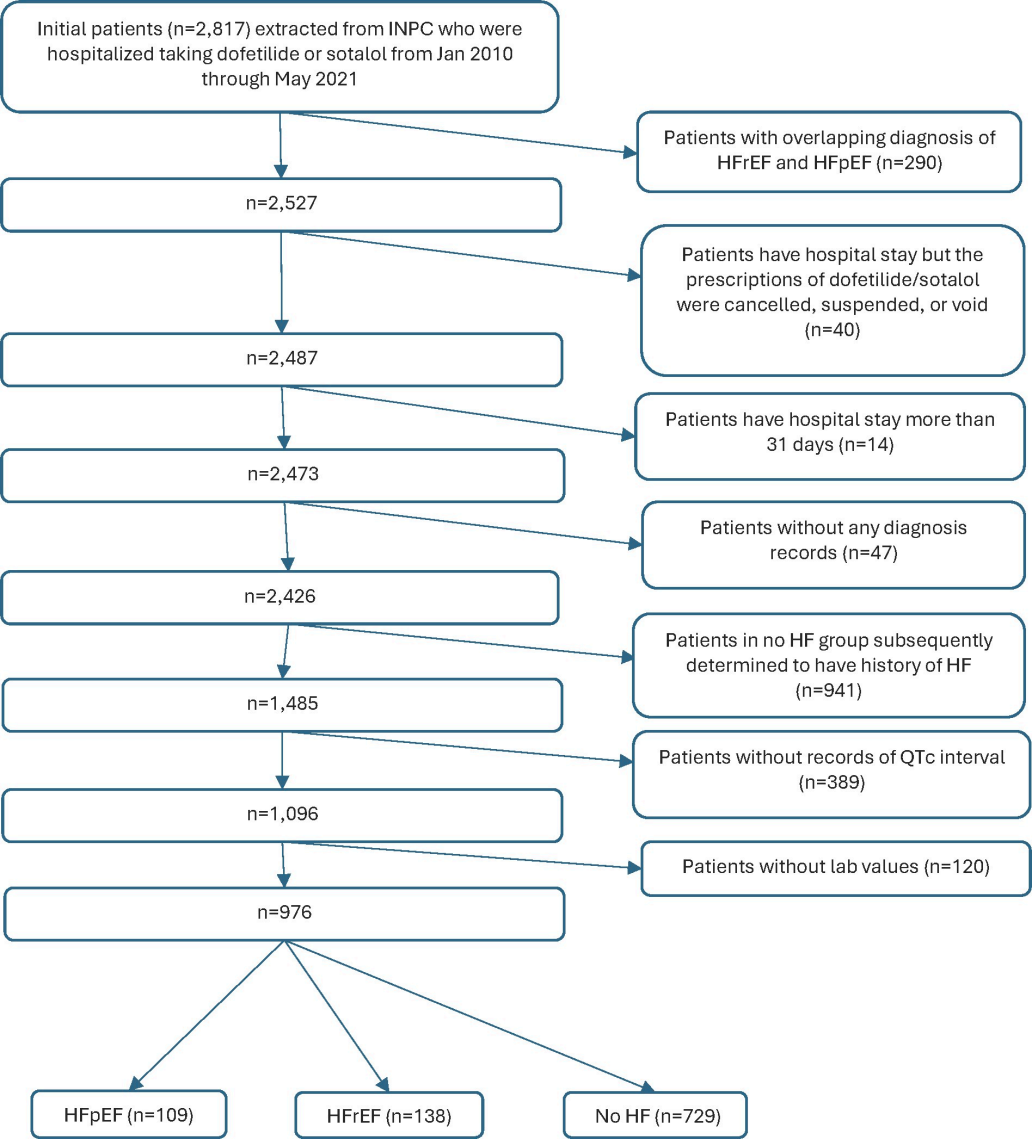
Flow diagram of INPC patient selection. INPC: Indiana Network for Patient Care; HF = Heart failure; HFrEF = Heart failure with reduced ejection fraction; HFpEF = Heart failure with preserved ejection fraction.

A total of 2,817 patients taking dofetilide or sotalol were identified (Fig 1). Of these, 976 patients met the inclusion criteria; 109 HFpEF patients, 138 HFrEF patients, and 729 patients without heart failure. One hundred fifty patients had two repeated hospitalizations (15 HFpEF, 14 HFrEF, 121 no HF), 27 patients had three repeated hospitalizations (2 HFpEF, 3 HFrEF, 22 no heart failure), and 23 patients without heart failure had ≥ 4 repeated hospitalizations. The median (IQR) hospital stay was 4 (3) days.

Patients’ baseline characteristics, laboratory values, concomitant drug use, and comorbidities are shown in Table 1. Most of the patients were White. A majority of HFpEF patients and individuals without heart failure were female, but the majority of patients with HFrEF were male. The distribution of comorbidities was significantly different across the three groups. The person-time average values of laboratory measurements and medication use are also listed in Table 1.

**Table 1:** Study cohort baseline characteristics*.

	HFpEF n = 109	HFrEF n = 138	No HF n = 729	p-value
Age (years)				
< = 60	11 (9.9)	48 (35.1)	183 (25.1)	0.001
61–70	29 (26.5)	30 (22.1)	227 (31.2)	0.001
71–80	45 (41.3)	40 (29.2)	215 (29.5)	0.001
>80	24 (22.3)	19 (13.6)	104 (14.3)	0.001
Median age (IQR), (years)	74 (14)	67 (20)	69 (17)	<0.001
Sex				
Female	67 (61.2)	43 (31.2)	330 (45.2)	0.11
Race				
White	105 (96.7)	124 (89.6)	691 (94.8)	0.66
Black	1 (0.8)	13 (9.1)	28 (3.8)	0.66
Other	3 (2.5)	2 (1.3)	11 (1.5)	0.66
Lab values†				
Mean serum potassium (SD) (mmol/L)	3.93 (0.59)	4.02 (0.60)	4.0 (0.50)	0.48
Mean serum creatinine (SD) (mg/dL)	0.98 (0.44)	1.05 (0.42)	0.89 (0.33)	<0.001
Hypokalemia (<3.5mmol/L)	16 (14.9)	16 (11.8)	54 (7.4)	0.002
Serum creatinine > 1.5 mg/dL	14 (3.1)	26 (18.8)	44 (6.1)	<0.001
Drug Use†				
Using other drugs known to cause TdP‡	26 (23.6)	41 (29.9)	206 (28.3)	0.39
Using drugs with a possible risk of TdP‡	50 (45.7)	74 (53.5)	398 (54.6)	0.067
Using drugs with a conditional risk of TdP‡	68 (62.1)	79 (56.9)	472 (64.7)	0.20
Calcium channel blockers	48 (44.3)	38 (27.6)	318 (43.6)	0.15
Loop diuretics	86 (78.6)	90 (65.5)	145 (19.9)	<0.001
Thiazide diuretics	5 (5.0)	8 (5.8)	67 (9.2)	0.04
Beta-blockers	51 (47.1)	87 (63.2)	332 (45.5)	0.04
ACE inhibitors	23 (21.4)	59 (43.1)	178 (24.4)	0.16
Angiotensin-receptor blockers	11 (10.0)	17 (12.6)	94 (12.9)	0.38
Digoxin	14 (12.9)	25 (17.8)	65 (8.9)	0.008
Aldosterone receptor antagonists	16 (15.0)	36 (25.9)	23 (3.1)	<0.001
Comorbidities				
Atrial Fibrillation	100 (92.1)	120 (86.9)	704 (96.6)	0.53
Hypertension	106 (97.5)	127 (92.2)	608 (83.4)	<0.001
Diabetes	52 (47.9)	59 (42.9)	233 (32.0)	<0.001
Chronic kidney disease	56 (51.2)	74 (53.3)	139 (19.1)	<0.001
Coronary artery disease	68 (62.0)	91 (66.2)	328 (45.0)	<0.001
Myocardial infarction	22 (19.8)	50 (36.4)	122 (16.8)	0.005
Hyperlipidemia	86 (78.5)	104 (75.3)	488 (66.9)	0.002
Stroke	13 (11.6)	17 (12.3)	107 (14.7)	0.26
COPD	55 (50.4)	50 (36.4)	166 (22.8)	<0.001

*Values are numbers (percentages) unless otherwise indicated.

†Time-dependent variables; shows the person-time average, at the time of dofetilide or sotalol initiation.

‡From the QT drugs list at www.crediblemeds.org ACE = Angiotensin converting-enzyme; COPD = Chronic obstructive pulmonary disease; IQR = Interquartile range; HFpEF: heart failure with preserved ejection fraction; HFrEF: heart failure with reduced ejection fraction; HF: heart failure; SD = Standard deviation; TdP = Torsades de pointes

### Proportion of patients with QTc > 500 ms during therapy with dofetilide or sotalol

The proportion of patients who developed QTc prolongation during therapy with dofetilide or sotalol was highest in the HFrEF group, followed by the HFpEF and control groups (Table 2). There are significant differences in QTc interval across HFpEF, HFrEF and no HF groups.

**Table 2:** Patients with QTc > 500 ms, 550 ms and 600 ms while taking dofetilide or sotalol during the hospital stay.

	HFpEF	HFrEF	No HF	P-value	Total
**QTc>500 ms, n (%)**	58 (53.2)	99 (71.7)	216 (30.0)	<0.001	373 (38.2)
**QTc> 550 ms, n (%)**	16 (14.7)	48 (34.8)	45 (6.17)	<0.001	109 (11.2)
**QTc > 600 ms, n (%)**	2 (1.8)	23 (16.7)	7 (1.0)	<0.001	32 (3.3)

HFpEF: heart failure with preserved ejection fraction; HFrEF: heart failure with reduced ejection fraction; HF: heart failure

### Unadjusted and adjusted odds of QTc interval prolongation

Table 3 shows the unadjusted and adjusted results from the GEE logistic regression. We found a significant positive association of HFrEF and HFpEF with QTc interval prolongation while taking dofetilide or sotalol during the hospital stay compared to patients with no heart failure (Table 3). Nonparametric bootstrapping with 10,000 replicates indicating model robustness yielded a similar result (Table 3). Table 4 shows the sensitivity analysis using backward selection incorporating variables with p<0.25 by GEE logistic analysis. We found significant positive associations of HFpEF and HFrEF with dofetilide/sotalol-associated QTc interval prolongation relative to patients with no HF. Nonparametric bootstrapping indicating model robustness yielded similar results (Table 4).

**Table 3:** Unadjusted and adjusted generalized estimating equations logistic regression model of factors associated with QTc interval >500 ms in hospitalized patients taking dofetilide or sotalol.

Group	Univariate	GEE Logistic model	GEE Bootstrapping
	OR (95% CI)	OR (95% CI)	OR (95% CI)
Disease group			
No HF	0.22 (0.16–0.31)	Ref	Ref
HFrEF	5.57 (3.63–8.56)	5.23 (3.15–8.67)	6.68 (3.83–11.15)
HFpEF	2.17 (1.41–3.35)	1.98 (1.17–3.33)	2.28 (1.31–3.72)
Age			
< = 60	0.98 (0.71–1.36)	Ref	Ref
61–70	0.98 (0.72–1.32)	1.18 (0.77–1.82)	1.28 (0.76–2.01)
71–80	1.21 (0.90–1.63)	1.22 (0.79–1.88)	1.70 (1.04–2.64)
>80	0.79 (0.54–1.15)	0.94 (0.56–1.57)	1.14 (0.63–1.92)
Sex			
Male	1.32 (1.00–1.75)	Ref	Ref
Female	0.75 (0.57–1.00)	0.91 (0.67–1.24)	0.96 (0.70–1.28)
Race			
White	0.97 (0.53–1.78)	Ref	Ref
Black	0.66 (0.31–1.43)	0.45 (0.19–1.04)	0.60 (0.17–1.37)
Other	2.82 (1.02–7.81)	2.34 (0.74–7.40)	2.93 (0.84–7.84)
Lab values			
Hypokalemia (<3.5mmol/L)	1.47 (0.92–2.33)	1.30 (0.81–2.11)	1.14 (0.51–2.17)
Elevated serum creatinine (>1.5 mg/dL)	1.77 (1.11–2.82)	1.21 (0.71–2.07)	1.16 (0.56–2.07)
Drug Use			
Using other drugs known to cause TdP*	0.73 (0.55–0.98)	0.79 (0.56–1.10)	0.71 (0.44–1.08)
Using drugs with a possible risk of TdP*	0.47 (0.36–0.61)	0.43 (0.31–0.59)	0.47 (0.29–0.72)
Using drugs with a conditional risk of TdP*	0.66 (0.51–0.87)	0.82 (0.59–1.14)	0.87 (0.53–1.37)
Calcium channel blockers	0.87 (0.65–1.15)	0.99 (0.72–1.37)	1.22 (0.74–1.88)
Loop diuretics	2.01 (1.50–2.70)	1.40 (0.99–2.00)	1.22 (0.67–1.99)
Thiazide diuretics	0.83 (0.51–1.35)	1.06 (0.62–1.81)	0.95 (0.42–1.83)
Beta-blockers	1.14 (0.87–1.50)	1.26 (0.92–1.73)	1.06 (0.65–1.66)
ACE inhibitors	0.93 (0.68–1.27)	0.75 (0.52–1.08)	0.84 (0.50–1.31)
Angiotensin-receptor blockers	1.13 (0.76–1.67)	1.17 (0.75–1.82)	1.30 (0.71–2.13)
Digoxin	1.01 (0.65–1.58)	0.87 (0.54–1.41)	0.74 (0.34–1.41)
Aldosterone receptor antagonists	1.86 (1.13–3.06)	0.90 (0.49–1.65)	1.37 (0.66–2.77)
Comorbidities			
Hypertension	1.05 (0.70–1.59)	0.76 (0.46–1.27)	0.67 (0.42–1.04)
Diabetes	1.25 (0.94–1.67)	1.17 (0.85–1.61)	1.16 (0.83–1.56)
Chronic kidney disease	2.00 (1.47–2.74)	1.34 (0.92–1.95)	1.36 (0.91–1.92)
Coronary artery disease	1.54 (1.16–2.03)	1.20 (0.86–1.69)	1.15 (0.82–1.57)
Myocardial infarction	1.69 (1.21–2.36)	1.40 (0.95–2.07)	1.45 (0.96–2.08)
Hyperlipidemia	0.99 (0.73–1.33)	0.75 (0.52–1.09)	0.74 (0.52–1.01)
Stroke	0.83 (0.56–1.23)	0.86 (0.57–1.29)	0.85 (0.54–1.25)
COPD	1.54 (1.13–2.09)	1.25 (0.89–1.77)	1.37 (0.98–1.85)

*From the QT drugs list at www.crediblemeds.org GEE = Generalized Estimating Equations; ACE = Angiotensin converting-enzyme; COPD = Chronic obstructive pulmonary disease; HFpEF: heart failure with preserved ejection fraction; HFrEF: heart failure with reduced ejection fraction; HF: heart failure; TdP = Torsades de pointes

**Table 4:** Sensitivity analysis: Unadjusted and adjusted generalized estimating equations logistic regression model with odds ratios for factors associated with QTc interval >500ms, backward selection of variables having p-value<0.25.

Group	Univariate	GEE Logistic model	Bootstrapping
	OR (95% CI)	OR (95% CI)	OR (95% CI)
Disease group			
No HF	0.22 (0.16–0.31)	Ref	Ref
HFrEF	5.57 (3.63–8.56)	5.23 (3.28–8.34)	6.30 (3.96–9.87)
HFpEF	2.17 (1.41–3.35)	1.90 (1.14–3.16)	2.19 (1.31–3.42)
Age			
< = 60	0.98 (0.71–1.36)	-	-
61–70	0.98 (0.72–1.32)	-	-
71–80	1.21 (0.90–1.63)	-	-
>80	0.79 (0.54–1.15)	-	-
Sex			
Male	1.32 (1.00–1.75)	-	-
Female	0.75 (0.57–1.00)	-	-
Race			
White	0.97 (0.53–1.78)	-	-
Black	0.66 (0.31–1.43)	0.46 (0.21–1.02)	0.56 (0.19–1.13)
Other	2.82 (1.02–7.81)	2.64 (0.85–8.18)	3.30 (1.03–8.67)
Lab values			
Hypokalemia (<3.5mmol/L)	1.47 (0.92–2.33)	-	-
Elevated serum creatinine (>1.5 mg/dL)	1.77 (1.11–2.82)	-	-
Drug Use			
Using other drugs known to cause TdP*	0.73 (0.55–0.98)	0.75 (0.54–1.04)	0.69 (0.42–1.05)
Using drugs with a possible risk of TdP*	0.47 (0.36–0.61)	0.42 (0.31–0.57)	0.47 (0.27–0.73)
Using drugs with a conditional risk of TdP*	0.66 (0.51–0.87)	-	-
Calcium channel blockers	0.87 (0.65–1.15)	-	-
Loop diuretics	2.01 (1.50–2.70)	1.40 (0.99–1.98)	1.25 (0.69–2.03)
Thiazide diuretics	0.83 (0.51–1.35)	-	-
Beta-blockers	1.14 (0.87–1.50)	1.25 (0.92–1.69)	1.05 (0.63–1.63)
ACE inhibitors	0.93 (0.68–1.27)	0.72 (0.51–1.02)	0.79 (0.46–1.25)
Angiotensin-receptor blockers	1.13 (0.76–1.67)	-	-
Digoxin	1.01 (0.65–1.58)	-	-
Aldosterone receptor antagonists	1.86 (1.13–3.06)	-	-
Comorbidities			
Hypertension	1.05 (0.70–1.59)	-	-
Diabetes	1.25 (0.94–1.67)	-	-
Chronic kidney disease	2.00 (1.47–2.74)	1.41 (0.99–2.01)	1.40 (0.97–1.91)
Coronary artery disease	1.54 (1.16–2.03)	1.23 (0.88–1.72)	1.15 (0.84–1.53)
Myocardial infarction	1.69 (1.21–2.36)	1.34 (0.91–1.97)	1.34 (0.90–1.89)
Hyperlipidemia	0.99 (0.73–1.33)	0.73 (0.52–1.02)	0.70 (0.51–0.95)
Stroke	0.83 (0.56–1.23)	-	-
COPD	1.54 (1.13–2.09)	1.26 (0.90–1.77)	1.37 (1.00–1.81)

*From the QT drugs list at www.crediblemeds.org. ACE = Angiotensin converting-enzyme; COPD = Chronic obstructive pulmonary disease; HFpEF: heart failure with preserved ejection fraction; HFrEF: heart failure with reduced ejection fraction; HF: heart failure; TdP = Torsades de pointes

## Discussion

While previous studies have shown that HFrEF is an independent risk factor for drug-induced QT-interval prolongation [4], this is the first study to investigate whether patients with HFpEF are also at increased risk. Dofetilide and sotalol are used commonly in patients with HFpEF due to the prevalence of AF in this population. These drugs are well-known to be associated with QT interval prolongation and TdP [2, 6, 28, 29], and QT prolongation and arrhythmia recurrence are the primary reasons for dofetilide and sotalol discontinuation [30–34]. Our results showed that HFpEF was associated with an increased risk of dofetilide/sotalol-associated QTc prolongation compared to patients who do not have heart failure. Our sensitivity analysis using backward selection and bootstrapping with 10,000 replicates showed a similar result. Our results also confirmed previous findings indicating that the odds of drug-induced QTc prolongation are higher in patients with HFrEF [4–13].

Previous studies have reported that HFpEF may be associated with lengthening of ventricular repolarization and associated arrhythmias. QT interval prolongation is present in up to 38% of patients with HFpEF [35]. QTc prolongation is predictive of diastolic dysfunction; in a study of patients undergoing echocardiography, the QTc was inversely associated with E’ velocity, QTc prolongation was associated with reduced E’ velocity, and QTc was the ECG measure most closely associated with reduced E’ velocity [18]. Cho et al studied 110 patients with HFpEF and 97 controls with normal diastolic function. QTc intervals were significantly longer in patients with HFpEF than in the control group (451±33 versus 432±29 ms, p<0.001). The risk of ventricular tachycardia was increased in patients with HFpEF (relative risk 2.86, 95% CI 1.15–7.08, p = 0.023), and there was a significant association between increased QTc interval and risk of ventricular tachycardia [36].

However, while these studies assessed lengthening of ventricular repolarization in patients with HFpEF, they did not assess the risk of drug-induced QTc prolongation or arrhythmias in this heart failure population. We previously conducted a small pilot study in which we enrolled 10 patients with HFpEF and 10 age- and sex-matched control subjects with no evidence of heart failure (control group). Patients with HFpEF demonstrated enhanced response to QT interval lengthening induced by ibutilide [22]. In the current study, we present further evidence that patients with HFpEF are at increased risk of drug-associated QTc prolongation. While the magnitude of increased risk of drug-induced QTc prolongation was not as high as that in patients with HFrEF, the risk of QTc prolongation associated with dofetilide and sotalol in patients with HFpEF was significantly increased compared to the reference group of patients with no heart failure. These data suggest that patients with HFpEF may be at heightened risk of drug-induced TdP and potentially sudden cardiac death; this hypothesis requires further investigation.

Mechanisms by which HFpEF may be associated with lengthening of ventricular repolarization have been investigated. In high-salt-fed rats developing echocardiographic-documented diastolic dysfunction with preserved ejection fraction and evidence of heart failure, Cho et al [20] reported that QTc intervals were significantly prolonged compared to control animals without HFpEF. In HFpEF rats, there was a high prevalence of ventricular arrhythmias and sudden cardiac death, which was associated with polymorphic ventricular tachycardia that degenerated into ventricular fibrillation. Rats with HFpEF displayed an increased susceptibility to complex ventricular arrhythmias (including polymorphic ventricular tachycardia or ventricular fibrillation) provoked by programmed electrical stimulation [21]. Single-cell recordings in ventricular myocytes from these hearts revealed prolongation of the action potential duration and downregulation of potassium currents including I_to_ (most prominently), I_Kr_ and I_K1_, and upregulation of I_CaL_. Both KCND3 transcript and Kv4.3 protein levels were significantly decreased in HFpEF rats compared with controls. Finally, the extent of cardiac fibrosis, measured by Masson trichrome staining, was also increased in HFpEF hearts, suggesting that both electrical and structural remodeling occur in these animals, promoting reentrant life-threatening ventricular arrhythmias [20].

In the 2023 American College of Cardiology/American Heart Association/American College of Clinical Pharmacy/Heart Rhythm Society guideline for diagnosis and management of AF, dofetilide and sotalol are recommended drugs for rhythm control [14]. In 2015, dofetilide and sotalol together comprised roughly 20% of all new antiarrhythmic drug prescriptions for AF [37]. From 2004–2016, prescriptions for antiarrhythmic drugs nearly tripled, and dofetilide and sotalol were among the drugs for which the most substantial increases in prescriptions occurred [38]. AF is common among patients with HFpEF, and therefore many HFpEF patients with AF may receive these antiarrhythmic agents.

Both dofetilide and sotalol are recommended for inpatient initiation, QT interval monitoring, and dose adjustment, to minimize the post-discharge risk of QT interval prolongation and TdP [14]. Despite this practice, however, there remains substantial proarrhythmic risk associated with these drugs following discharge. In one analysis, one-year mortality was significantly higher in patients with HFrEF who continued dofetilide following the inpatient initiation period compared to patients in whom dofetilide was discontinued during inpatient initiation [39]. Patients who had TdP during the inpatient initiation period had higher 1-year mortality than those who did not. Between 1–17 months following inpatient initiation, 6% of patients with hypertrophic cardiomyopathy (mean left ventricular ejection fraction 63%) taking sotalol and 10% taking dofetilide developed QT interval prolongation [40]. Therefore, while the inpatient initiation of dofetilide and sotalol is intended to minimize longer-term risk of QT prolongation and proarrhythmia, this risk remains substantial during outpatient therapy, particularly in patients with HFrEF or HFpEF.

In our study, the incidence of QTc interval prolongation during inpatient initiation of dofetilide and sotalol was relatively high. In some previous studies, while the incidence of patients requiring discontinuation of dofetilide or sotalol due to QTc prolongation during inpatient initiation was reported, the overall incidence of QTc prolongation was not reported [5, 41, 42], precluding comparison with our data. However, in an analysis of 379 patients undergoing inpatient initiation of dofetilide or sotalol, 36% of whom had heart failure (proportion of HFrEF versus HFpEF not reported), 45% of patients developed QTc interval > 500 [30]. This is a higher incidence than in our control group of patients with no heart failure, and close to the incidence in our patients with HFpEF. In a population of older adults (> 80 years of age), of whom roughly ½ had heart failure (proportion of HFpEF versus HFrEF not reported), 68% of patients developed QTc > 500 ms during inpatient initiation of dofetilide and sotalol [43]. This is a higher incidence of QTc prolongation than in our population (though similar to that in our HFrEF patients), perhaps because of the older age of the patients. Overall, the incidence of QTc prolongation in our study seems similar to that reported in previous studies.

Limitations of this study warrant consideration. Data from the INPCR database were from hospitalized patients taking dofetilide or sotalol. Therefore, we do not know if patients developed QT interval prolongation after discharge. In addition, the days of hospitalization were somewhat variable, so it is possible that patients with longer hospital stays may have higher odds of developing QTc interval prolongation. However, we attempted to minimize differences by offsetting the GEE model’s coefficients of days in hospitals. Despite receiving therapy with dofetilide or sotalol, not all patients in our database have daily ECG data during hospitalization. This may have occurred for patients who were not being initiated on dofetilide or sotalol as inpatients, but rather were taking dofetilide or sotalol but were hospitalized for other reasons. It is therefore possible that we may have missed QTc prolongation that occurred on those days without recorded ECGs. Moreover, for many patients in the database, there were no baseline QT intervals, so we cannot rule out whether QTc prolongation was pre-existent for some patients. However, this seems unlikely, because initiation of dofetilide or sotalol is contraindicated if patients have pre-existing QTc interval prolongation [24, 25], and during dofetilide or sotalol initiation, dose titration is performed based on QTc intervals. While we have serum creatinine data in the database, we do not have estimated creatinine clearances, and therefore our metric of kidney function is solely serum creatinine. Death records were not available in this dataset, and this limits our ability to study QTc interval-associated death. Moreover, we could not compare the odds of adverse cardiac outcomes (TdP or other ventricular tachycardia or sudden cardiac arrest) by comparing HFrEF or HFpEF to no HF patients because there were very few recorded occurrences. We also found, as an unexpected incidental paradoxical finding, that the concomitant use of drugs with a “possible” risk of TdP (based on the QT drugs list from www.crediblemeds.org) was associated with a lower risk of QT prolongation. Reasons for this paradoxical finding are unclear. Finally, due to a substantial amount of missing data for serum calcium and magnesium concentrations reported in the INPCR database, we were not able to include serum calcium/hypocalcemia and serum magnesium/hypomagnesemia as a factor in the analysis.

## Conclusions

Patients with HFpEF are at increased risk of QT interval prolongation associated with dofetilide and sotalol compared to patients who do not have heart failure. Further study is needed to determine if the risk of QT interval prolongation associated with other QT-prolonging drugs is also enhanced in patients with HFpEF and whether patients with HFpEF are at increased risk of drug-induced TdP and/or sudden cardiac death.

## Supporting information

S1 Table ICD-9 CM and ICD-10 CM codes used to define HFpEF, HFrEF, patients without HF, cardiovascular-related disease, and comorbidities from Indiana Network for Patient Care Research database. (DOCX)
