# Supplementary material for: Drug-induced QT interval prolongation in patients with heart failure with preserved ejection fraction
Source: PLoS One. 2024 Aug 19;19(8):e0308999. doi: 10.1371/journal.pone.0308999 (PMC11332914; doi:10.1371/journal.pone.0308999)
Supplement: S1 Table — (DOCX) [file pone.0308999.s001.docx]

## **Supporting Information**

## **Table S1**. ICD-9 CM and ICD-10 CM codes used to define HFpEF, HFrEF, patients without HF, cardiovascular-related disease, and comorbidities from Indiana Network for Patient Care

|  | ICD-9 CM Definition | ICD-10 CM Definition |
| --- | --- | --- |
| HFrEF | 428.2 | I50.2 |
| HFpEF | 428.3 | I50.3 |
| Congestive heart failure | 398.91, 402.01, 402.11, 40.291, 404.01, 404.03, 404.11, 404.13, 404.91, 404.93, 425.4, 425.5, 425.7, 425.8, 425.9, 428 | I099, I110, I130, I132, I255, I420, I425, I426, I427, I428, I429, I43, I50, P290 |

| Comorbidities | | |
| --- | --- | --- |
| Hypertension | 401, 402, 403, 404, 405 | I10, I11, I12, I13, I15 |
| Chronic kidney disease | 585 | N170, N171, N172, N178, N179, N180, N188, N189, N19, N990 |
| Coronary artery disease | 411, 413, 414 | I251, I253, I254, I255, I256, I257, I258, I259 |
| Myocardial infarction | 410, 412 | I21, I22, I252 |
| Hyperlipidemia | 2722, 2724 | E782, E784, E785 |
| Stroke | 430, 431, 432, 4321, 4329, 43301, 43311,43321, 43331, 43381, 43391, 43401, 43411, 43491, 435, 436 | H341, I60, I61, I63, I64, G450, G451, G452, G453, G458, G459 |
| Chronic obstructive pulmonary disease | 491, 492, 496 | J43, J44, J45 |

CM = Clinical Modification; HFpEF: Heart failure with preserved ejection fraction; HFrEF: Heart failure with reduced ejection fraction; ICD = International Classification of Diseases;
